# Copper sulfide nanoribbon growth triggered by carbon nanotube aggregation *via* dialysis†

**DOI:** 10.1039/d2ra04832a

**Published:** 2022-11-02

**Authors:** Tomomi Tanaka, Misaki Kurihara, Makoto Kuwahara, Shota Kuwahara

**Affiliations:** Department of Chemistry, Faculty of Science, Toho University 2-2-1 Miyama Funabashi 274-8510 Chiba Japan syouta.kuwahara@sci.toho-u.ac.jp; Graduate School of Engineering and Institute of Materials and Systems for Sustainability, Nagoya University Chikusa Nagoya 464-8603 Japan

## Abstract

The growth of copper sulfide (Cu_*x*_S) nanoribbons, a class of Cu_*x*_S nanomaterials, was achieved by the aggregation of single-walled carbon nanotubes (SWCNTs) *via* a dialysis process. The obtained nanoribbon structure and its constituent elements on a film of SWCNT aggregates were confirmed by transmission electron microscopy (TEM) and scanning transmittance electron microscopy–energy dispersive X-ray spectroscopy. The subsequently obtained TEM images and Raman spectra revealed that nucleus synthesis and subsequent growth of Cu_*x*_S nanoribbons occurred during the aggregation of SWCNTs. The growth procedure described in this work provides an approach for the wet chemical synthesis of metal sulfide nanomaterials.

## Abstract 2

The growth of copper sulfide (Cu_*x*_S) nanoribbons, a class of Cu_*x*_S nanomaterials, was achieved by the aggregation of single-walled carbon nanotubes (SWCNTs) *via* a dialysis process.

## Graphical abstract

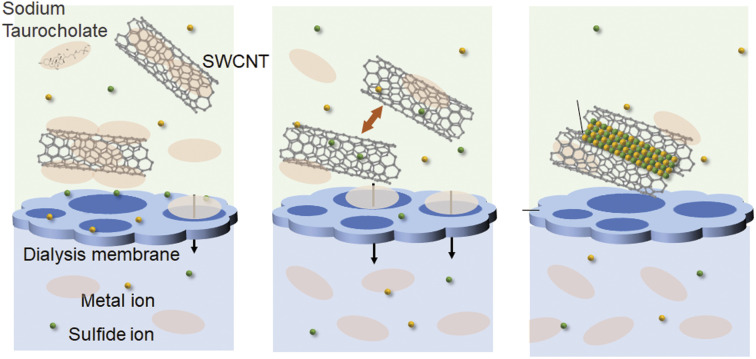

## Introduction

The electronic, magnetic, and optical properties of nanomaterials change depending on the constituent elements and shapes of these materials. The intrinsic properties of nanomaterials arise as a result of boundary conditions due to dimensional restriction, resulting in a variety of properties that are not found in bulk materials. Recently, one-dimensional (1D) and two-dimensional (2D) nanomaterials, such as nanotubes,^1–3^ graphene^4,5^ and transition metal dichalcogenide monolayers,^6–9^ have been intensively explored as promising materials for electronics and optoelectronics. Achieving homogeneous growth of 1D and 2D nanomaterials with high quality and a large area is important for advancing the use of these nanomaterials, and various techniques can be used: mechanical or chemical exfoliation of bulk single crystals, chemical vapor deposition (CVD)^6,8,10^ and molecular beam epitaxy.^11,12^ Unlike 1D and/or 2D nanomaterials, nanoribbons, a type of pseudo-1D nanomaterial, are also considered as dimension-restricted materials being affected by width-induced confinement and edge states, which result in their intrinsic physical properties.

Copper sulfate (Cu_*x*_S), which is known as a p-type semiconductor, has been utilized for the application of catalysts,^13^ solar cells^14–16^ and Li ion batteries^17,18^ due to its good electrical and optical properties and good physical and chemical stability.^19^ The nanoscale morphology of Cu_*x*_S expands its potential applications:^20^ nanoparticles for bioimaging and photothermal therapy with near-infrared absorption by modulating their size,^21–23^ nanosheets or flower-like morphology for Na^+^ ion batteries.^24,25^ Morphology-controlled growth of Cu_*x*_S has been achieved *via* various techniques, such as sonochemical,^26,27^ hydrothermal,^15,28,29^ solvothermal^19,30^ and CVD methods,^31^ to advance the application of Cu_*x*_S.

In this study, we grew Cu_*x*_S nanoribbons, another nanostructure class of Cu_*x*_S, by the aggregation of coexistent single-walled carbon nanotubes (SWCNTs) *via* dialysis. Transmission electron microscopy (TEM) observations revealed that the formation of a Cu_*x*_S nucleus upon aggregation of SWCNTs triggers the growth of Cu_*x*_S nanoribbons with lengths greater than 500 nm by using sulfide and copper impurities inside the dialysis membrane. We also examined the correlation between the growth of Cu_*x*_S nanoribbons and the progression of the aggregation of SWCNTs by sequential TEM observation and Raman analysis.

## Results and discussion

### TEM and EDS characteristics of Cu_*x*_S nanoribbon

**Fig. 1:**
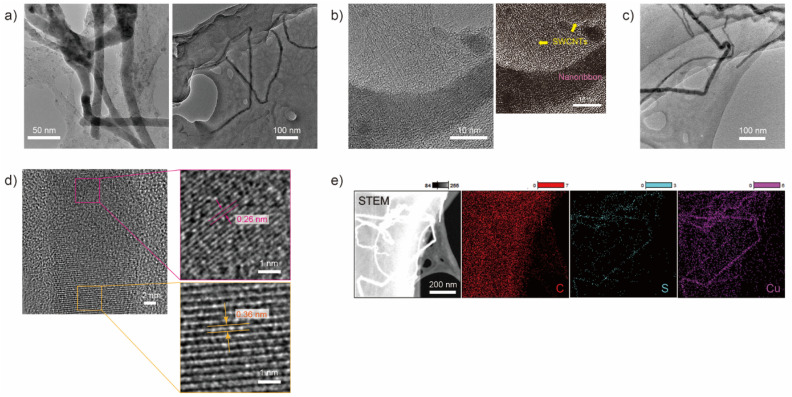
(a)–(d) TEM and HRTEM images showing nanoribbon structures on the aggregation of SWCNTs. (e) High angle dark-field-STEM image and EDS mapping of the spatial distribution of C, S and Cu of the nanoribbons and the aggregation of SWCNTs in the region shown in (c).

After dialysis for 48 hours, SWCNTs aggregated inside a dialysis tube. The obtained sample was characterized by using TEM as shown in Fig. 1. A film-like morphology, which has a different contrast from the supporting membrane of the grid, was observed, and a ribbon-like morphology with dark contrast was formed on the film (Fig. 1(a)). The ribbon-like structure was 14.5 ± 1.6 nm wide and over 500 nm long, and consisted of a straight growth region and flexed points. The ribbon-like structure was observed within the region of the film-like morphology and consisted of SWCNTs aggregates with a diameter of approximately 1 nm, as determined by high-resolution TEM (HRTEM) as shown in Fig. 1(b).

An HRTEM image of the ribbon-like structure observed in Fig. 1(c) is shown in Fig. 1(d). In the lower half of the dark contrast region, a lattice pattern with an interlayer spacing of approximately 0.36 nm is observed, while a pattern with an interlayer spacing of 0.26 nm is observed in the top half. These lattice patterns cannot be explained by a regularly or randomly stacked SWCNT array and do not match the lattice pattern of graphite, one of the possible contamination of an SWCNTs sample.

Next, we performed scanning TEM (STEM)-energy dispersive X-ray spectroscopy (EDS) measurements to map the constituent elements of the ribbon-like structure as shown in Fig. 1(e). The EDS map revealed that the obtained structure consists essentially of copper (Cu) and sulfur (S), while carbon (C) was detected in a whole film-like structure corresponding to SWCNT aggregation. Other candidate constituent elements include cobalt (Co) and molybdenum (Mo), which were used as catalytic metals for SWCNT synthesis. The energy of the X-ray spectral features related to Mo was indistinguishable due to overlap with the features related to S. However, Mo is assumed to be scattered in the film-like structure region since Co was detected in the same region as C. X-ray photoelectron spectroscopy also showed the existence of S and Cu in the sample (Fig. S1†), although the characteristic peaks of Cu and S were not clearly observed due to small amounts of constituents of the nanoribbon in a sample. Note that the lattice patterns, 0.36 nm and 0.26 nm, observed in an HRTEM image shown in Fig. 1(d) seem to be assigned as the (203) plane of Cu_2_S^32^ and the (104) plane of CuS,^25^ respectively. The further investigation of the localized composition of the nanoribbion structure is needed to prove the assignment. S and Cu were possibly derived from the impurities of the dialysis tube without pretreatment (Fig. S2†), and in the pretreated dialysis tube that was free from impurities, the ribbon-like structure with a lattice pattern was not observed (Fig. S3†).

### SWCNT dependence on the growth of Cu_*x*_S nanoribbon

**Fig. 2:**
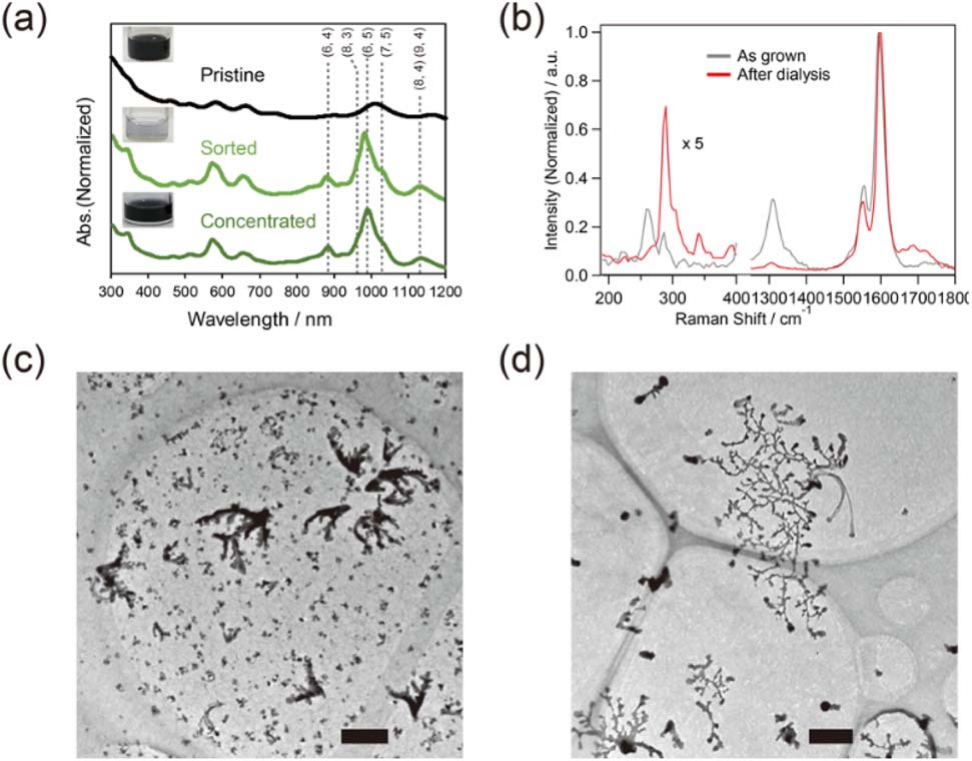
(a) Optical absorption spectra of the sorted and concentrated SWCNTs. The black, light green, green lines correspond to the pristine, sorted and concentrated SWCNTs, respectively. (b) Raman spectra of the as-grown SWCNTs (gray) and aggregated SWCNTs after dialysis (red) at a wavelength of 633 nm. TEM images of the samples obtained after dialysis (c) with and (d) without sorting of the SWCNTs. Scale bar: 500 nm.

SWCNTs in film-like structures are thought to serve as a surface for Cu_*x*_S nanoribbon growth. Structurally homogeneous SWCNTs were applied to trigger the growth of the nanoribbon and were compared with as-grown SWCNTs. The absorption in the ranges of 500–700 nm and 850–1100 nm, which corresponded to S_22_ and S_11_ of semiconducting SWCNTs (s-SWCNTs) with a small diameter, respectively, increased after sorting (Fig. 2(a)), and the radial breathing mode (RBM) of SWCNTs in the Raman spectra in the range of 200–270 cm^−1^ was diminished (Fig. 2(b)), indicating that small-diameter s-SWCNTs were selectively obtained in the sorted sample.^33,34^ The ratio of the signal intensity of the D-band to the G-band decreased from 0.3 to 0.04 before and after the dialysis process, suggesting the removal of amorphous carbon *via* the sorting and dialysis processes. The Cu_*x*_S nanoribbon successfully grew on the aggregation of the sorted SWCNTs, while the grain-like structure of Cu_*x*_S was obtained *via* SWCNT dispersion without sorting. It was suggested that structurally homogeneous SWCNTs or the aggregation of less unwanted substances, such as amorphous carbon, promote the growth of Cu_*x*_S nanoribbons due to smooth sliding of the crystal nucleus of Cu_*x*_S on the aggregation surface. The sorted SWCNTs were used for subsequent experiments.

### Sequential TEM observation and Raman analysis on the growth of Cu_*x*_S nanoribbon

**Fig. 3:**
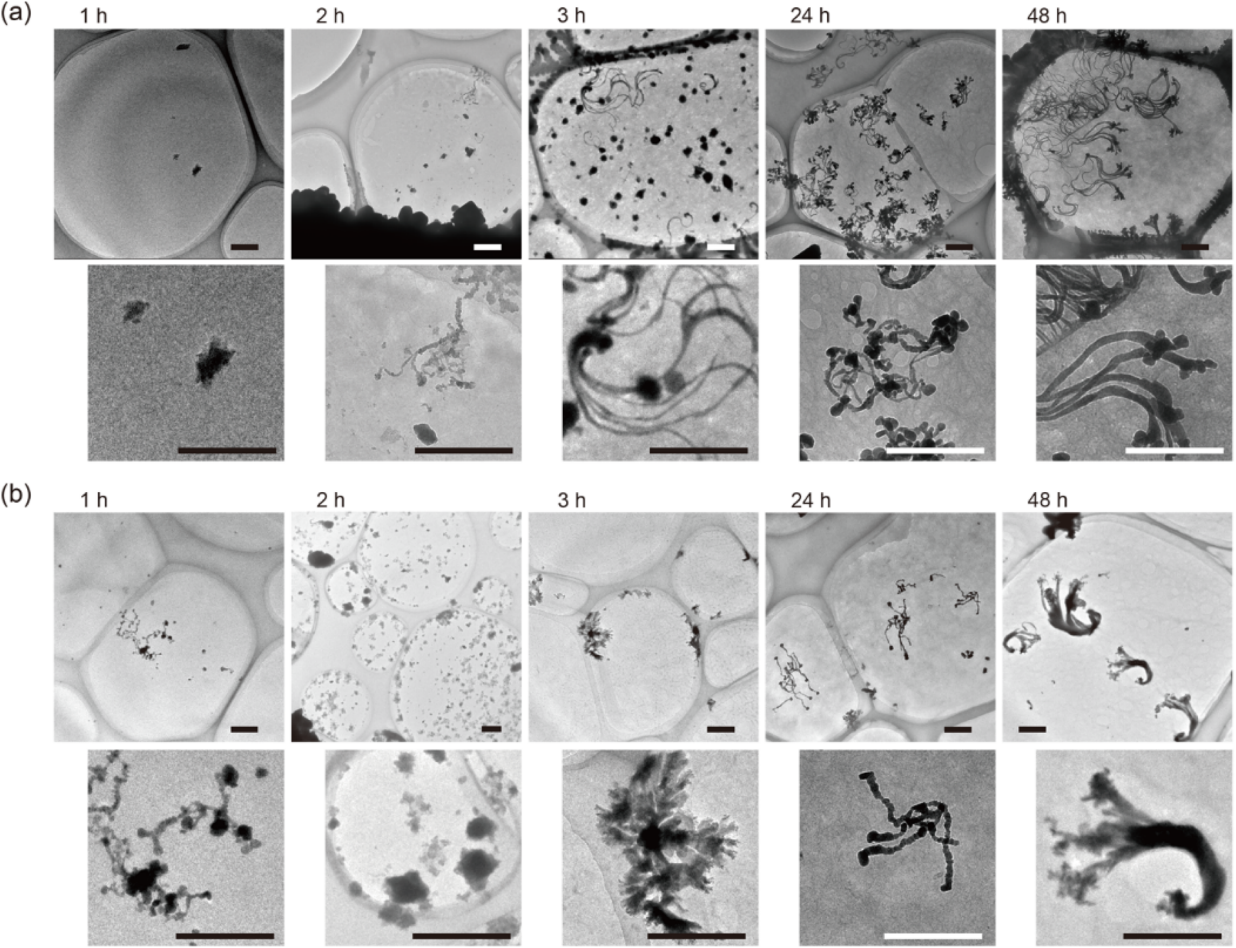
The sequentially obtained TEM images of samples after dialysis for each time with a dialysis tube of (a) MWCO: 3500 and (b) MWCO: 12 000–14 000. Scale bar: 500 nm.

The growth of Cu_*x*_S nanoribbons was tracked by changing the time for the dialysis process, and each obtained sample was examined by TEM observation, as shown in Fig. 3. In the samples obtained after 1 h and 2 h of dialysis, particles for the nucleus of Cu_*x*_S nanoribbons growth were observed on the SWCNT aggregation; subsequently, nanoribbons started to grow after 3 h and continued to grow after 24 h. In a dialysis tube with different molecular weight cutoff (MWCO) values, the same growth behavior was observed, as shown in Fig. 3(b). The number of particles observed on the sample increased with increasing MWCO size, suggesting that rapid exhaust of constituents in the SWCNT dispersion inside a dialysis tube increased the rate of aggregation of SWCNTs as well as the nucleus synthesis.

**Fig. 4:**
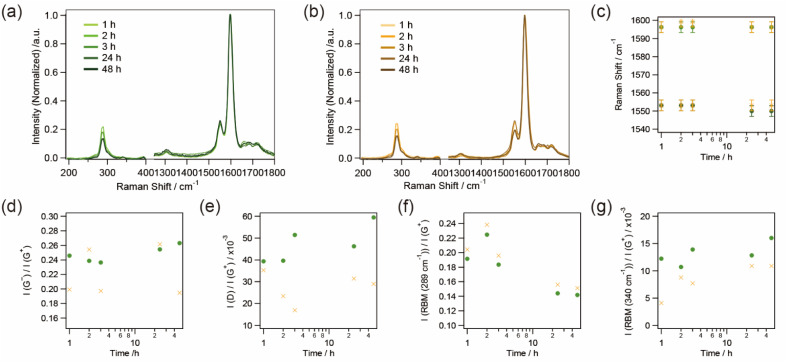
Raman spectra upon aggregation of SWCNTs at different dialysis times in dialysis tubes with MWCO values of (a) 3500 and (b) 12 000–14 000. Dialysis time dependence of the Raman spectra of the SWCNT aggregates in a dialysis tube with MWCO values of 3500 (green square) and 12 000–14 000 (orange cross): (c) peak shifts of the G^+^ and the G^−^ bands, (d) the ratio of peak intensity of the G^−^ band to the G^+^ band, (e) the ratio of peak intensity of the D band to the G^+^ band, (f) the ratio of peak intensity of the RBM band at 289 cm^−1^ to the G^+^ band, and (g) the ratio of peak intensity of the RBM band at 340 cm^−1^ to the G^+^ band.

The aggregation of SWCNTs for the support of Cu_*x*_S nanoribbons was evaluated by sequential Raman spectra of dialyzed samples with different MWCO tubes (Fig. 4(a) and (b)). A peak at 289 cm^−1^ corresponding to (7,5) nanotubes^35^ was observed in the RBM region since s-SWCNTs with small diameters were contained in the sorted samples. The positions of the G^+^ (1590 cm^−1^) and the G^−^ (1550 cm^−1^) bands as well as their full width at half maximum (FWHM) values fluctuates within a resolution of ∼3 cm^−1^, suggesting that the probability of charge doping to SWCNTs from Cu_*x*_S nanoribbons was very small. The ratio of the intensity of the D-band as well as the G^−^ band to that of the G^+^ band was also not correlated with the time for dialysis. These results indicate that SWCNTs in the sample aggregated and formed a film-like structure without changes in their composition of surface conditions.

The ratio of the intensity of the RBM to that of the G^+^ band, however, changed with the time of dialysis. As the dialysis time increased, the peak intensity at 340 cm^−1^, corresponding to (6,4) nanotubes,^35,36^ gradually increased, whereas that at 289 cm^−1^ decreased. In the dispersion of SWCNTs, the peak of (7,5) nanotubes was thought to be resonant with a wavelength of 633 nm (1.96 eV) due to the *E*_22_ optical transition of 1.92 eV while that of (6,4) nanotubes with an *E*_22_ optical transition of 2.13 eV was off-resonant.^37^ However, the aggregation of SWCNTs, which changes in the circumstance of SWCNTs from surfactant-micelles to SWCNTs with the same electronic structure, seems to slightly modulate the density of states of each SWCNT,^38^ resulting in the band-gap energy corresponding to a decrease in the *E*_22_ optical transition of the (6,4) nanotubes upon aggregation of the SWCNTs. As a result, the peak at 340 cm^−1^ gradually became resonant with the excitation wavelength of 633 nm while that at 289 cm^−1^ became off-resonant. The ratio of the intensity of the RBM to that of the G^+^ band started to change in 2–3 h of the dialysis time, and corresponds to the growth of Cu_*x*_S nanoribbons observed by TEM as shown in Fig. 3. The sequentially obtained TEM images and Raman spectra suggest the following growth mechanism of Cu_*x*_S nanoribbons: (1) aggregation of SWCNTs as surfactants are removed, (2) nucleus synthesis of Cu_*x*_S, (3) growth of Cu_*x*_S nanoribbons with ongoing aggregation and packing of SWCNTs.

## Experimental

### Method

The basic procedure for the dispersion and sorting of SWCNTs *via* the phase transition of PNIPAM has been described in previous reports.^33,34^ All chemical were used as received. Briefly, a 9 mg portion of SWCNTs (CoMoCAT; CG100, ≥ 89% carbon basis, diameter: 0.7–1.3 nm, Sigma-Aldrich, USA) was dispersed in 10 g of Milli-Q water containing 2.0 wt% sodium cholate (SC; for biochemistry, FUJIFILM Wako Pure Chemical Corp., Japan) or sodium taurocholate (STC; Nacalai Tesque Inc., Japan) using a probe tip ultrasonicator (TOMY, UR-20R, Japan) for 1.5 h in a cold bath. A 500 μL aliquot of the SWCNT dispersion and 110 μL of a diluted NaClO (active chlorine of 8.5–13.5%; Nacalai Tesque Inc., Japan) aqueous solution with a dilution factor of 0.06 were mixed in a vial by means of a vortex mixer for 5–20 minutes, which was followed by mixing with 2 mL of a 5 wt% PNIPAM (avg. MW: 40 000, Sigma-Aldrich, USA) aqueous solution. The prepared solution was mixed again with the vortex mixer, subsequently heated at 45 °C and incubated for 15 min. After the phase transition, the liquid phase was collected. The collected sample was then concentrated by using an ultrafiltration technique with a membrane with a molecular weight cutoff (MWCO) of 100 000 (Amicon® Ultra Centrifugal Filters Ultracel®, Millipore Sigma-Aldrich) at 14 000 × *g* for 10 minutes.

A 50 μL aliquot of the SWCNT dispersion or the concentrated sample was used for the following dialysis procedure. The surfactant, STC or SC, was removed from the obtained sample through dialysis in 500 mL of Milli-Q water using a Visking membrane (MWCO: 3500, 12 000–14 000, cellulose tube). The Milli-Q water for dialysis was exchanged at 1, 2, 3, 24 and 48 hours after the start. Each obtained sample then cast on a grid with a porous support membrane for subsequent TEM observation. For dialysis free from both heavy metals and sulfide in the cellulose tube, a pretreated dialysis tube (Spectra/Por®7 Biotech RC Tubing, MWCO: 3.5–5 kD, FED) was used without further treatment.

### Characterization

High-resolution TEM images were obtained on an electron microscope at acceleration voltages of 120 kV (Tecnai G2 F20 S-TWIN, FEI, Thermo Fisher Scientific, USA) and 200 kV (JEM-ARM200F, JEOL, Japan). To map the constituent elements in a sample, STEM-EDS characterization was performed on an EX-24221M1G5T equipped in a JEM-ARM200F (JEOL, Japan) at an acceleration voltage of 200 kV. X-ray photoelectron spectroscopy was carried out on an AXIS Nova (Kratos Analytical, SHIMADZU Corp., Japan). Optical absorption spectra of the obtained samples were collected on a UV-vis-NIR spectrophotometer (UV-3600, Shimadzu Corp., Japan). Raman spectra of the obtained samples on a grid for TEM observation were collected *via* a homemade Raman setup with a QE65 Pro spectrometer (Ocean Optics) and a He–Ne laser with a wavelength of 633 nm. Scanning electron microscopy (SEM) and EDS were performed on an electron microscope at an acceleration voltage of 20 kV (S-3500N, Hitachi, Japan, and EMAX300, Horiba, Japan).

## Conclusions

The growth of Cu_*x*_S nanoribbons was found to be triggered by the aggregation of SWCNTs *via* dialysis of surfactant-wrapped SWCNTs. The nanoribbon structure was measured to be 14.5 ± 1.6 nm wide and over 500 nm long by TEM observations, and the element composition was confirmed by STEM-EDS mapping. The obtained Cu_*x*_S structure constitutes another category of Cu_*x*_S nanomaterials grown by wet chemical process. The sequentially obtained TEM images and Raman spectra reveal that nucleus synthesis and the subsequent growth of Cu_*x*_S nanoribbons occurred during the formation of well-packed aggregates of SWCNTs inside a dialysis tube. Although there are issues in controlling the composition of metallic sulfide and its growth length and width by the concentration of precursors, Cu_*x*_S nanoribbons are expected to have unique nanostructure-based applications and to add value because of their pseudo-one-dimensional characteristics.

## Conflicts of interest

There are no conflicts to declare.

## Supplementary Material

RA-012-D2RA04832A-s001
