# Supplementary material for: Copper sulfide nanoribbon growth triggered by carbon nanotube aggregation via dialysis
Source: RSC Adv. 2022 Nov 2;12(48):31363–8. doi: 10.1039/d2ra04832a (PMC9627581; doi:10.1039/d2ra04832a)
Supplement: RA-012-D2RA04832A-s001 [file RA-012-D2RA04832A-s001.pdf]

Electronic Supplementary Material (ESI) for RSC Advances.
This journal is © The Royal Society of Chemistry 2022

Supporting Information for

Copper Sulfide Nanoribbon Growth Triggered by Carbon Nanotube Aggregation via Dialysis

XPS characterization of a sample after dialysis.

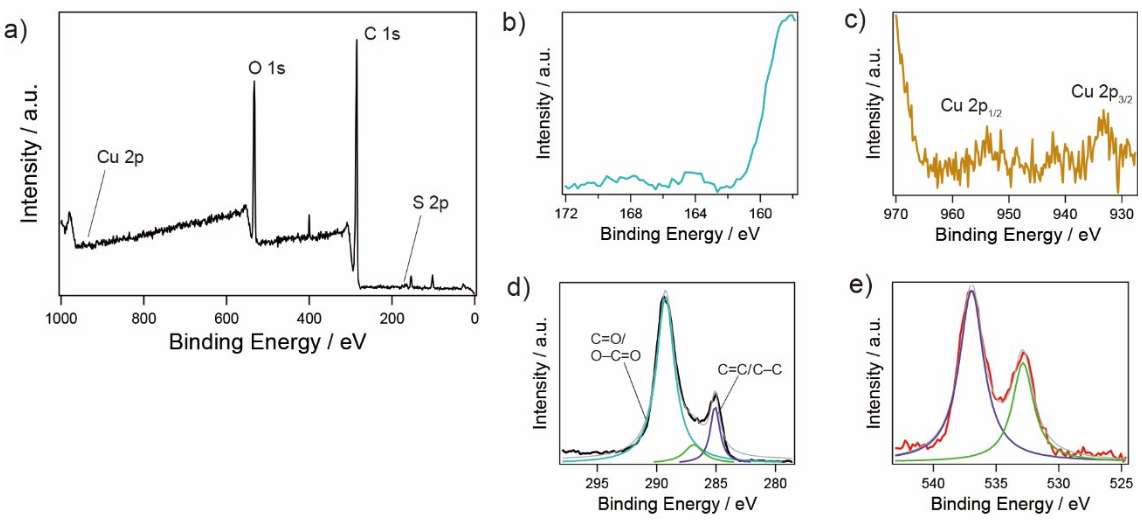

Figure S1. (a) XPS analysis and high resolution XPS spectrum of (b) S 2p, (c) Cu 2p, (d) C 1s and (e) O 1s for a sample obtained after dialysis. The obtained C-O peak in the XPS data is understood to be derived from the defect of SWCNTs or unremoved thermoresponsive polymer, the residues of the sample.

SEM image and EDS measurement of a dialysis tube.

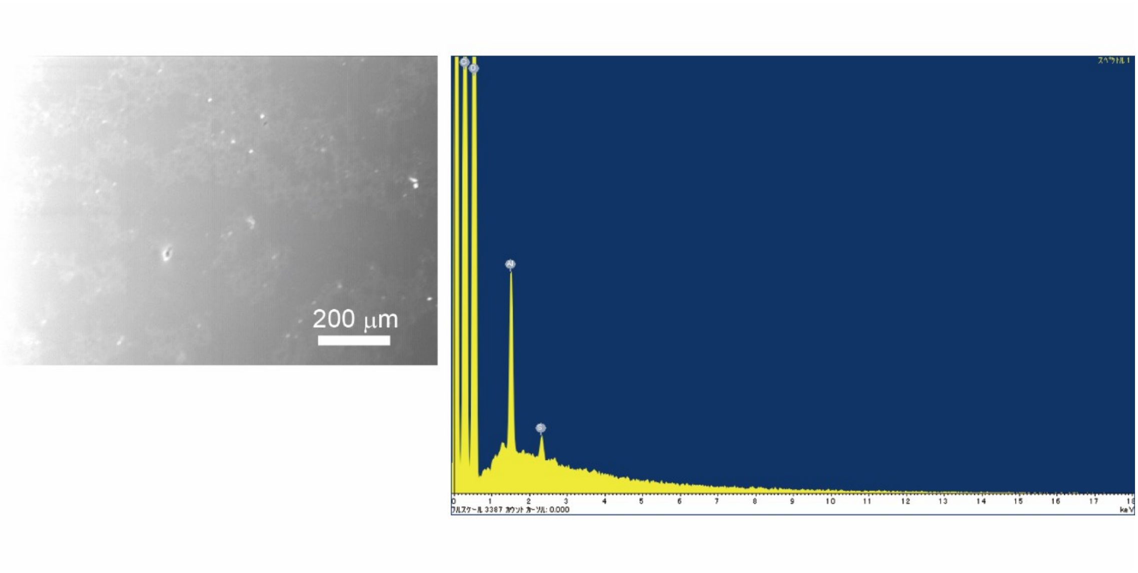

Figure S2. SEM and EDS of the inside of a dialysis tube and a Visking membrane (MWCO: 3,500, cellulose tube). The EDS peak appearing at 2.3 keV corresponds to S Kα. The peak attributed to Al is background due to the base material used.

TEM images of samples after dialysis for with a pretreated dialysis tube.

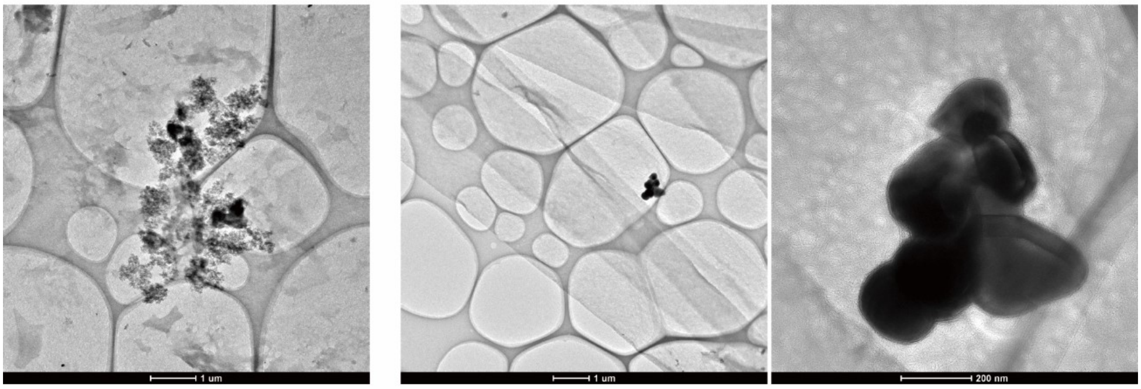

Figure S3. TEM images of samples after dialysis for 48 h with a pretreated dialysis tube (Spectra/Por®7 Biotech RC Tubing, MWCO: 3.5-5 kD).
